# Circadian clocks, cognition, and Alzheimer’s disease: synaptic mechanisms, signaling effectors, and chronotherapeutics

**DOI:** 10.1186/s13024-022-00537-9

**Published:** 2022-05-07

**Authors:** Kari R. Hoyt, Karl Obrietan

**Affiliations:** 1 Division of Pharmaceutics and Pharmacology, Ohio State University, 412 Riffe Building, 12th Ave, Columbus, OH 43210 USA; 2 Department of Neuroscience, Ohio State University, Graves Hall, 333 W. 10th Ave, Columbus, OH 43210 USA

**Keywords:** Alzheimer’s disease, Circadian, Suprachiasmatic nucleus, Memory, Cortex, Limbic system, Chronotherapeutics

## Abstract

Modulation of basic biochemical and physiological processes by the circadian timing system is now recognized as a fundamental feature of all mammalian organ systems. Within the central nervous system, these clock-modulating effects are reflected in some of the most complex behavioral states including learning, memory, and mood. How the clock shapes these behavioral processes is only now beginning to be realized. In this review we describe recent findings regarding the complex set of cellular signaling events, including kinase pathways, gene networks, and synaptic circuits that are under the influence of the clock timing system and how this, in turn, shapes cognitive capacity over the circadian cycle. Further, we discuss the functional roles of the master circadian clock located in the suprachiasmatic nucleus, and peripheral oscillator populations within cortical and limbic circuits, in the gating of synaptic plasticity and memory over the circadian cycle. These findings are then used as the basis to discuss the connection between clock dysregulation and cognitive impairments resulting from Alzheimer’s disease (AD). In addition, we discuss the conceptually novel idea that in AD, there is a selective disruption of circadian timing within cortical and limbic circuits, and that it is the disruption/desynchronization of these regions from the phase-entraining effects of the SCN that underlies aspects of the early- and mid-stage cognitive deficits in AD. Further, we discuss the prospect that the disruption of circadian timing in AD could produce a self-reinforcing feedback loop, where disruption of timing accelerates AD pathogenesis (e.g., amyloid deposition, oxidative stress and cell death) that in turn leads to a further disruption of the circadian timing system. Lastly, we address potential therapeutic approaches that could be used to strengthen cellular timing networks and, in turn, how these approaches could be used to improve cognitive capacity in Alzheimer’s patients.

## Background

The circadian timing system is an evolutionarily conserved cell-autonomous process that creates a daily rhythm with a period of approximately 24 h. Within mammals this clock timing process is distributed through all organ systems and most cell types, and recent work has revealed that the timing properties of these cellular clocks is regulated by the entraining effects of the master circadian oscillator located in the suprachiasmatic nucleus (SCN) of the hypothalamus.

**Fig. 1:**
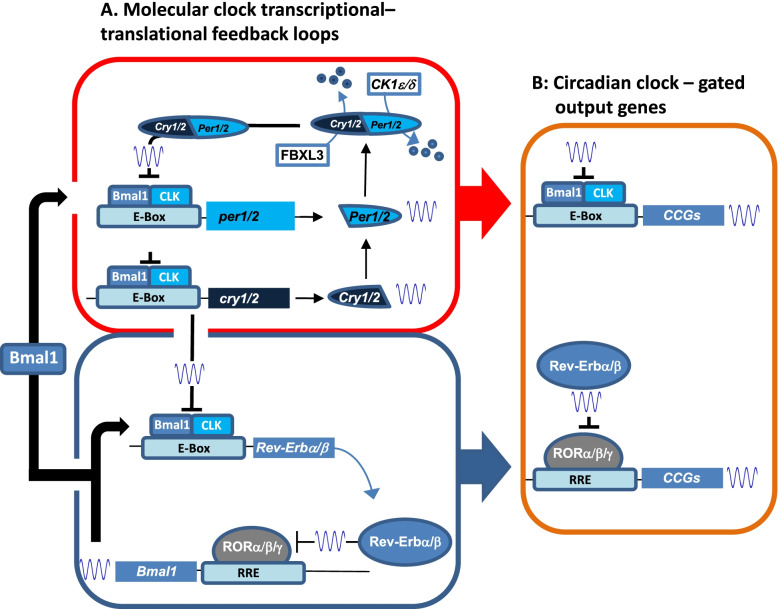
Transcription feedback loops that form the basis of the circadian timing system. The red box denotes the feedback loop centered on the rhythmic expression of period and cryptochrome. The blue box denotes the feedback loop centered on the rhythmic expression of *Bmal1* and *Rev-Erbα/β*. The orange box denotes rhythmic drive that is conferred to core clock-regulated genes (CCGs) via the Bmal1/Clock complex, and via the competitive interaction between *Rev-Erbα/β and* RORα/*β*/γ

To begin to understand the fundamental architecture of the circadian timing system, it is instructive to start with a deconstruction of timekeeping within the SCN at a molecular, cellular and systems level. The SCN is composed of approximately 10,000 GABAergic neurons. If one were able to peer inside an SCN neuron and monitor the mechanisms that underlie the circadian timing system, they would observe an interlocking set of transcriptional and post-translational feedback/regulatory processes that are centered on the daily oscillation in the expression of the *period* (*per1* and *per2*) and *cryptochrome* (*cry1* and *cry2*) genes. The rhythmic regulation of *per* and *cry* gene transcription is driven via an E-box-binding heterodimeric basic helix-loop-helix transcription factor formed by CLOCK (or NPAS2: [1]) and BMAL1 (brain and muscle ARNT-like protein 1, also called MOP3) [2, 3]. As their cytoplasmic concentrations rise PER and CRY proteins dimerize and translocate to the nucleus, where they function as potent negative regulators of CLOCK-BMAL1-mediated transcription. A daily release from the repressive effects of PER/CRY is mediated by the progressive phosphorylation of PER1/2 by casein kinase 1ε and δ, which tags PER proteins for ubiquitin-targeted degradation [4, 5], and by CRY1/2 protein degradation via the SCF/Fbxl3 ubiquitin ligase complex [6, 7]. One full cycle of PER and CRY expression (transcriptional activation followed by feedback repression) defines the circadian period. Consistent with this idea, genetic deletion of *Bmal1* or the double deletion of *Cry1/Cry2*, or *Per1/Per2* leads to clock arrythmia [8–10]. Further the length of the ‘circadian’ cycle can be regulated by affecting the functional properties of CK1 or FBXL3 [11]. For example, gain-of-function mutations that increase CK1 activity, destabilizes PER1/2, thus resulting in a shortening of the clock period [5, 12]; conversely, pharmacological approaches that reduce CK1 activity results in a slowing of the clock transcriptional feedback loop [13]. Output from this Clock/Bmal1-driven transcriptional feedback loop underlies the expression of circadian clock-controlled output genes; in fact, across the body, organ-specific profiling has shown that ~ 43% of the transcriptome is under the control of this transcriptional circuit [14] (Fig. 1).

In addition to this primary clock timing loop, an interlocking secondary transcriptional feedback loop is centered on the rhythmic expression of transcription repressors *Rev-erbα* (*Nr1d1*) and Rev-erbβ (*Nr1d2*), which compete for binding at the retinoic acid-related orphan receptor (ROR)-response element (RRE) with the transcriptional activators RORα, RORβ and RORγ. These primary and secondary transcriptional loops intersect at two points: 1) CLOCK and BMAL1 driving the rhythmic expression of *Rev-erbs*, and 2) rhythmically expressed Rev-erbs competing with RORs at RREs in the promoter of *Bmal1*, which underlies the daily rhythm in *Bmal1* expression [15, 16]. The disruption of Rev-erbα/β expression (via both gene knockout- and knockdown-based approaches) leads to loss of *Bmal1* rhythms and a marked disruption in clock timing (both within the SCN and in peripheral oscillator populations) [16, 17]. In addition, through the direct clock-regulated rhythmic drive at the RRE, Rev-erbα/β have been shown to play a major role in regulating rhythmic expression of clock controlled genes, including those that play a role in metabolism and inflammation [18] (Fig. 1).

Consistent with the idea that the SCN serves as the master clock, the selective disruption of SCN timing, either through tissue lesioning-based approaches, or through the genetically-based abrogation of the core clock transcriptional feedback loop, leads to a loss of clock timing properties (i.e., circadian arrythmia), which manifests at the level of both basic systems-level physiology, including melatonin release, adrenal corticosterone output and core body temperature as well as behavioral processes, including locomotor activity, and sleep [19–25]. Together, these observations support the long-standing idea that the SCN functions as the master pacemaker.

**Fig. 2:**
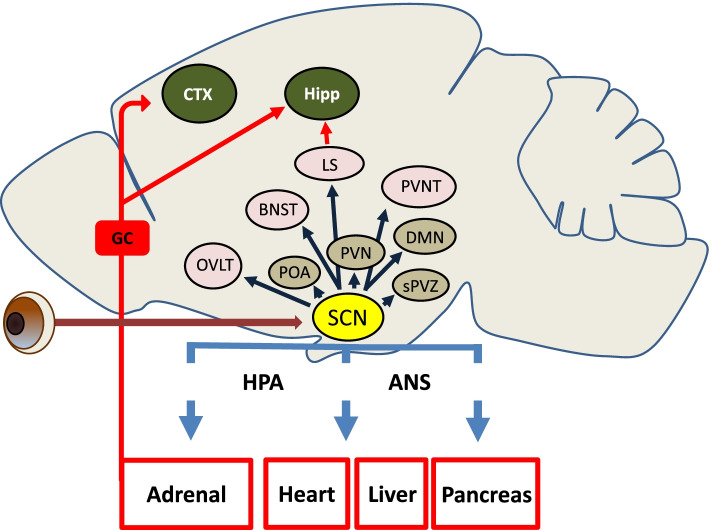
The SCN master clock: major efferents within the CNS, and clock-gated peripheral organ systems. Black arrows denote direct synaptic targets of the SCN. Red arrows denote cortico-limbic brain regions that are under the indirect control of the SCN, either via output from the lateral septal area (LS), or via glucocorticoid (GC) release from the adrenal gland. Blue arrows denote SCN output via the hypothalamic pituitary axis (HPA) and the autonomic nervous system (ANS) that gates the inherent oscillatory capacity of peripheral organs. The brown arrow denotes the direct, monosynaptic, input to the SCN from the retina. sPVZ: subparaventricular zone; PVNT: paraventricular nucleus of the thalamus; BNST: bed nuclei of the stria terminalis; OVLT: organ vascular of lamina terminalis; POA: preoptic area; PVN: paraventricular nucleus; DMN: dorso-medial nucleus; Hipp: hippocampus; CTX: cortex

Clock timing cues that emanate from the SCN are relayed via efferents that project largely within the hypothalamus (e.g., to the paraventricular nucleus, dorso-medial nucleus, preoptic area, and the subparaventricular zone), with limited projections to extrahypothalamic targets, including the paraventricular nucleus of the thalamus, bed nuclei of the stria terminalis, the vascular organ of lamina terminalis and the lateral septal area [26–30] (Fig. 2). These projection pathways have been implicated in the clock regulation of diverse physiological processes, including, sleep, melatonin synthesis, feeding, reproduction, memory and even aggressive behavior [31–34].

Through its effects on pituitary output and the autonomic nervous system, the SCN imparts a daily rhythm on the physiological properties of most, if not all, peripheral organ systems [35, 36]. For example, a daily rhythm in sympathetic nerve tone underlies the circadian rhythm in cardiovascular activity (e.g., blood pressure and heart rate) and respiratory function; likewise, through both pituitary and autonomic output, the SCN clock gates the release of glucocorticoids from the adrenal cortex [37]. Confirmation of the central role of the SCN in peripheral organ rhythms has been shown in a number of studies where SCN lesioning and SCN-targeted core clock gene deletion approaches lead to a damping and, ultimately, a loss of circadian output from peripheral organs [38–41]. Notably, this loss of clock-gated output is thought to largely result from a desynchronization of peripheral oscillator populations: as such, the SCN plays a key role in maintaining robust, entrained rhythms in peripheral organ oscillator populations [42, 43].

### Circadian timing in the CNS, with an emphasis on cortical and limbic regions

Beyond the SCN, clock timing properties have been detected throughout the CNS in both neuronal and non-neuronal cell populations. For example, protein profiling, along with RNA-based profiling have shown that rhythms in core clock genes including *period* and *cryptochrome* genes are detected within neocortical and limbic structures (i.e., the hippocampus and amygdala) [44–48]. Further, recent in vivo based imaging that utilized a *cry*-driven fluorescent protein approach revealed that hippocampal CA1 neurons exhibit marked oscillatory capacity [49]. Consistent with the idea that the SCN regulates oscillator capacity within the CNS, a number of studies have shown that the rhythm-generating ability of cortical and limbic circuits requires input, in the form of an entrainment cue, from the SCN. For example, Rath, et al. showed that the rhythmic expression of core clock genes within the cortex is lost when the SCN is lesioned [46]. Interestingly, the output signal from the SCN that drives the rhythm generating capacity of cortical and limbic circuits, appears to be mediated by a combination of synaptic projections from the SCN to the lateral septum [50], and an SCN-driven daily oscillation in glucocorticoids (GC) that are released from the adrenal gland. With respect to the role of GC in cortical rhythms, several studies have shown that adrenalectomy in rats result in a damping/loss of clock gene rhythms within the cortex and limbic structures [51–53]; further, the pharmacological inhibition of the NR3C1 glucocorticoid receptor disrupted the daily rhythm in hippocampal LTP [54]. The process by which rhythmic release of GC drives clock gene rhythms appears to be mediated, at least in part, via a transcriptional mechanism in which GC drives glucocorticoid responsive element- (GRE) mediated expression of core clock genes [55]. Whether the induction of core clock genes by GC regulates circadian rhythms by enhancing cellular oscillatory capacity or by maintaining the entrainment of cellular clocks is not known. Thus, the rhythm in GC appears to be a key conduit by which the SCN provides a daily timing cue to cortical and limbic circuits. Notably, this GC-based clock gene oscillator model does not appear to be specific to the CNS; rather, a number of studies have shown that GC play a key role in maintaining clock rhythms and entrainment in a number of peripheral organ systems [55–57]. Interestingly, the phasing of forebrain clocks can also be influenced by time cues that function independently from the SCN. Along these lines a ‘misaligned’ feeding schedule, in which mice are allowed access to food only during the daytime, led to an inversion of the phasing of hippocampal oscillators (i.e., per2 levels peaked during the daytime rather than the night) [58, 59], whereas no effect on SCN clock phase was observed [58]. Collectively, these data indicate that 1) the SCN clock is critical for robust system-wide forebrain rhythms, and 2) the SCN does not have an ‘iron-grip’ over forebrain oscillators-as such, this opens-up forebrain clocks to an array of physiological (and potentially, pathophysiological) influences that could alter the underlying functional properties of cortical and limbic circuits.

### Clock gating of cognition

Numerous studies in both invertebrate and vertebrate model systems have shown that cognitive processing is gated over the circadian cycle. For example, the clock has been shown to modulate the efficiency of memory formation and recall [60–63]. Further, memory retrieval is disrupted when the temporal organization of clock timing is compromised. Along these lines, shifting of the light/dark cycle, which leads to the disruption/desynchronization of the circadian timing system, triggers a marked deficit in the recall of spatial tasks and in the retention/retrieval of active and passive avoidance tasks [64–67]. Given the large number of reviews that have comprehensively described the connection between clock timing and cognition at a systems and behavioral level, here we will focus on the possible cellular mechanism(s) by which the clock could modulate learning and memory over the circadian cycle. As was noted, circadian pacemaker activity is a distributed process, where the rhythm generating capacity of the SCN sets the phasing of ancillary oscillator populations within cortical and limbic circuits that underlie learning and memory. The distributed nature of clock rhythms raises a question: do these forebrain clocks play a critical role in the gating of cognitive capacity, or, is output from the SCN sufficient to drive daily rhythms in cognition? To examine this question, studies over the past several years have utilized a combination of anatomical lesioning and clock gene deletion methods to assess the contributions of the SCN, as well as cortical and limbic clocks, to the daily gating of memory.

Initially, it is worth briefly discussing the complex and somewhat contradictory results that have come from SCN-based lesioning approaches. Work by Phan et al. (2011) reported that SCN ablation in C57/Bl6 mice triggered deficits in contextual fear memory and spatial memory [68], and Shimizu et al. (2016) found that SCN lesioning disrupts recognition memory [69]. In contrast, a number of studies in rodent models have reported that SCN ablation does not profoundly affect learning or memory [70–73], and, surprisingly, some studies have reported that SCN ablation actually improves memory [74]. Clock gene knockout studies, which render animals arrhythmic, have also reported varying degrees of cognitive deficits, depending on the targeted gene(s) and the memory test [75–78].

These conflicting results suggest that there are missing elements in our understanding of the functional contribution of the SCN to the cellular and systems processes that underlie learning and memory. Within this context, the work of Ruby et al., which employed a Siberian hamster model in which the SCN pacemaker can be rapidly and irreversibly rendered arrhythmic via a simple manipulation of the lighting cycle has been particularly informative. In this approach, which side-steps the complicating effects of SCN lesioning (e.g., damage to the hypothalamic tissue surrounding the SCN), and clock gene deletion approaches that result in marked developmental and health issues [79], arrhythmic hamsters with intact SCN exhibited a complete loss of spatial and recognition memory capacity (assessed using novel object recognition and spontaneous alternation assays). Interestingly, this effect was reversed, and normal cognitive capacity was restored when the arrhythmic SCN tissue was surgically ablated [50, 73]. Collectively these studies indicate that dysregulated output from the SCN (i.e., output that does not generate a robust entrainment/time cues) leads to a marked disruption of cognition.

One of the most direct approaches to addressing the role of non-SCN clocks in memory formation is to use targeted gene disruption methods. In this approach, the functionality of the molecular clock is abrogated in the neuronal circuits that underlie learning and memory, while the functional properties of the SCN remain intact. Using this strategy, both Snider et al. (2016; 2018) and Shimizu et al. (2016) selectively deleted the *Bmal1* gene from excitatory neurons of the forebrain, including those within the cortex and hippocampus, while not affecting *Bmal1* expression in the SCN, and thus retaining the clock timing properties of the master oscillator [69, 80, 81]. Loss of *Bmal1* led to deficits in hippocampal-dependent measures of spatial memory acquisition and the time-of-day dependent-modulation of novel object recognition memory, thus supporting the idea that extra-SCN timing within forebrain circuits is critical for clock-gating of cognition. In something of a parallel to these experimental approaches, Hasegawa et al., (2019) used a transgenic approach to drive the expression of a dominant-negative form of Bmal1 in a tetracycline-inducible manner within cortical and limbic circuits, but not in the SCN. Overexpression of this construct led to the disruption of clock timing properties within the hippocampus, and the disruption of hippocampal memory retrieval [82]. The idea that ancillary clocks of the CNS function in coordination with the SCN clock to modulate cognition was further supported by a recent study in which a transgenic CK1ε *Tau* chimeric mouse model was used to create a discordant period between the SCN clock (~ 24 h) and extra-SCN clocks of the CNS (~ 20 h). Memory performance using the novel object location test revealed circadian misalignment between the SCN oscillator and forebrain closks led to an inability of chimeric mice to effectively discriminate novel from familiar objects [83]. Together, these data, along with the SCN lesioning and conditional extra-SCN clock knockout strategies outlined above indicate that the SCN functions in a coordinated manner with cortical and limbic system clocks to shape the efficiency of learning and memory over the 24 h cycle.

### Clock gating of hippocampal and cortical physiology

A key question here relates to how the circadian timing system could shape the cellular physiology of the synaptic circuits that underlie cognition. One of the most straightforward ways in which this could occur would be for the clock to modulate cellular excitability. In this model, the capacity of a set level of synaptic input required to actuate intracellular signaling events that underlie short- or long-term plasticity, would vary as a function of the circadian cycle; hence the response properties of the postsynaptic cell would vary according to clock time.

This model is based on a large literature showing that the TTFL regulates SCN neuronal membrane excitability, including the membrane potential and action potential generation, as a function of circadian time. This process is mediated, in part, through a complex interplay of rhythmic changes in the expression and function of channels, receptors, and transporters. These changes, which are comprehensively reviewed in Harvey et al., and Colwell et al., include a daily rhythm in the expression of voltage-dependent and independent Na^+^, Ca^2+^ and K^+^ channels that regulate the firing properties of SCN neurons over the circadian cycle [84, 85].

Within cortico-limbic circuits, work from several studies support the idea that the clock shapes the efficacy of cellular excitability and ultimately synaptic plasticity. In the earliest in vivo work from the hippocampus, Barnes et al. (1997) revealed that the response of granule cells to entorhinal afferent input varies as a function of circadian time in both nocturnal (rats) and diurnal (monkeys) species [86]. Interestingly several studies in humans also support the idea that cortical excitability is modulated by the circadian timing system [87, 88, 89], and that this daily rhythm is critical for normal cognition. Consistent with this idea, aging-related decreases in the daily rhythm in cortical excitability are associated with decreased executive performance and reduced cognitive flexibility [88]. Interestingly, transcranial magnetic stimulation-based cortical profiling studies in humans have shown that the circadian clock modulates GABA-mediated cortical inhibition [89, 90]. Of note, the daily rhythm in cortisol levels appears to play a key role in the modulation of GABA-mediated inhibition [89, 90]. Further, prior sleep history was not correlated with this daily rhythm in GABA-mediated intracortical inhibition, and sleep deprivation did not alter this rhythm, thus indicating that the circadian clock underlies GABAergic inhibition [89].

Long-term potentiation (LTP), which is a well-accepted cellular model of memory formation [91], has also been shown to be under the control of the circadian timing system. Harris and Teyler were the first to report hippocampal LTP is modulated over the circadian cycle, with the CA1 cell layer showing enhanced levels of potentiation during the circadian day relative to the circadian night [92]; conversely, within the granule cell layer potentiation was greater during the circadian night than during the circadian day. These studies have been followed by a large number of reports that have systematically examined electrophysiological properties of LTP that are affected by the circadian timing system. Notably, in C57/Bl6 mice, Chaudhury et al. (2005) found that the circadian clock regulated CA3 Schaffer collateral-evoked LTP in the CA1 cell layer [93]. Interestingly, analysis of the current-response relationship (which can be a reflection of a change in the sensitivity of neurons to excitatory input) revealed that the magnitude of the stimulus-evoked input/output function was greater during the circadian night than during the circadian day in CA1 pyramidal neurons, and the rate of LTP decay was reduced during the night relative to during the day. Similar time-of-day variations in cellular excitability have been described by a number of groups, using a number of methods and model systems. Along these lines, depolarization-evoked excitability has been shown to peak during the night in the CA3 cell layer [94], and time-of-day differences in evoked responses have also been observed in the dentate gyrus (an effect that was, in part, ascribe to the effects of extracellular adenosine) [95]. These data reveal an underlying set of cellular and synaptic processes by which the circadian clock can shape the functional properties of neuronal circuits that underlie learning and memory. Further, these data also raise the prospect that these effects on cellular excitability could be reflected in the response profiles of intracellular signaling pathways that underlie synaptic plasticity.

### Cellular and molecular neuronal plasticity and the circadian clock

With respect to molecular and cellular mechanisms by which the circadian timing system could shape cognitive capacity, several reports merit discussion.

Recent work by Hasegawa et al. (2019) found that circadian timekeeping capacity within the hippocampus plays a key role in memory retrieval via the daily modulation of dopamine-dependent signaling [82]. In this paper, the authors used a transgenic approach in which a CaMKII-tTA line was used to drive the expression of a dominant-negative form of Bmal1 within the excitatory neuronal populations of the cortex and limbic system. In this mouse model, damped clock oscillations were shown to lead to a reduction in time-of-day-dependent memory retrieval, as assessed using a social recognition task, novel object recognition test, and a contextual fear conditioning task. In all of these tasks, time-of-day retrieval, rather than time-of-day memory encoding was compromised. These effects were shown to be associated with a downregulation of dopamine D1 and D5 receptor expression, type 1 adenylyl cyclase and A-kinase Anchor Protein 5 (AKAP5). In total, these results point to a reduction in clock-gated G-protein-dependent cAMP formation. Consistent with this idea, the authors showed that the administration of rolipram or injection of the D1/5R agonist SKF38393 rescued the retrieval deficit in dominant-negative BMAL1 mice. Together these data suggest that the daily/circadian modulation of memory retrieval is driven in part by the capacity of the circadian clock to gate the efficacy of dopamine-dependent cAMP/PKA signaling.

Additionally, work from our lab found that a daily rhythm in the microRNA miR132 shapes cognitive capacity over the circadian cycle. microRNAs are a class of small non-coding transcripts that function as negative regulators of mRNA translation. Through the modulation of mRNA translation, microRNAs have been shown to affect activity-dependent neuronal plasticity, and in turn complex cognitive processing [96–98]. Our work found that miR-132 is rhythmically expressed under the control of the circadian clock in the cortex and hippocampus. Further, using a combination of conditional knock-out and tetracycline-inducible mouse models, we found that constitutive expression of miR132 (i.e., suppressing miR132 rhythms) blocked time-of-day dependent memory recall (assessed using contextual fear conditioning and novel object location paradigms) [99].

Rhythmic regulation of ERK activity and cAMP production has been shown to play a role in the clock-gating of cognition. Along these lines Eckel-Mahan et al., [100] identified a daily rhythm in ERK activity and cAMP levels within the hippocampus, and found that the disruption of rhythmic ERK activity, via the deletion of calcium-sensitive adenylyl cyclases, constant light treatment or the pharmacological disruption of MAPK signaling, led to the disruption of clock-gated contextual memory formation and persistence. Interestingly, building off this finding, studies by Rawashdeh et al. reported that the MAPK target pP90RSK accesses the cellular nucleus by dimerization with Period1 and that this interaction is associated with the daily rhythm in hippocampal plasticity and memory [101]. Further, work by Shimizu found that SCOP (suprachiasmatic nucleus circadian oscillatory protein) underlies rhythmicity of MAPK signaling within the cortex and hippocampus, and that the daily rhythm of ERK activity in the hippocampus was disrupted in SCOP conditional KO animals. Of note, at a mechanistic level, SCOP inhibits MAPK signaling by sequestering nucleotide-free Ras [102], and the dynamic circadian regulation of RAS/MAPK signaling via SCOP has been shown to be mediated by a time-of-day accumulation of SCOP within membrane rafts, where it most effectively binds to RAS [100].

Glycogen synthase kinase 3 (GSK3) has also been shown to function as a clock-gated regulator of synaptic plasticity in the forebrain. GSK3 is a highly expressed serine/threonine-specific kinase, with a large number of target proteins (> 100) [103], including a number of proteins that play a key role in the core clock timing process [104–107]. Consistent with this, GSK3 has been shown to have profound effects on the phasing and periodicity of the core clock oscillator. Along these lines, suppression of GSK3 activity results in an increase in rhythm amplitude and period shortening of the core clock feedback loop [108–110]. Further, inhibition of GSK3β results in a phase delay of the core clock oscillator, whereas GSK-3β overexpression advances the period of the core clock oscillator [111]. With respect to clock-regulation of neuronal plasticity, Besing et al. found that GSK3 plays a key role in the daily rhythm of hippocampal LTP generation [110]. In specific, pharmacological inhibition of GSK3 led to a reduction in the magnitude of LTP specifically during the night time domain, and this effect correlated with a marked rhythm in GSK3β phosphorylation. The cumulative effects of these daily changes in activation/response potential of these intracellular signaling pathways could be reflected in the daily gating of the system-level response properties of cortico-limbic circuits, and this, in turn could manifest as a daily rhythm in cognitive capacity.

Finally, to provide a bit of context, and to shore-up the rationale for the concepts outlined above, it is worth noting that a number of these clock-gated hippocampal cellular plasticity pathways (e.g., ERK/MAPK signaling, miR132 expression, cAMP levels) are under the control of the circadian clock in the SCN. For example, within the SCN, MAPK pathway activity is tightly regulated; hence the clock gates the capacity of the MAPK pathway to be activated. The best example of this phenomenon is the response properties of the pathway to photic stimulation. Along these lines, photic stimulation during the circadian night triggers MAPK pathway activation, as assessed by monitoring the activation state of the MAPK effector kinase ERK, whereas exposure to the same stimulus during the circadian day does not trigger ERK activation [112, 113]. The effects of ERK activation during the circadian night in the SCN are profound; light-evoked MAPK activation regulates the resetting of the SCN oscillator. Further, these effects correlate with the time-of-day gated induction of plasticity associated immediate early genes, including *cFos*, *JunB*, and *EGR1* (along with the induction of the core clock gene *Period1*) [113, 114].

Returning to the concept of the gating of signaling pathways within limbic circuits, it is worth noting that the effects of the circadian clock need not impose tight binary, SCN-like, gating over kinase pathway activity to confer time-of-day modulation over learning and memory. Rather, the effects might be more subtle, as one would expect given the modulatory nature of clock timing on cognition (again, this is in contrast to the tight, time-domain-delimited, control that the SCN clock imparts over kinase response properties). As yet, it is unclear whether similar or distinct time-of-day-dependent gating mechanisms regulate kinase activation in the SCN and in cortical-limbic oscillator populations. Clearly this is a line of inquiry that merits extensive investigation.

### Clock dysregulation and Alzheimer’s disease

Alzheimer’s disease (AD) is a complex neurodegenerative disorder that is functionally characterized by a deterioration of cognitive abilities, which, often times, initially manifests as a disruption in short-term memory. As the disease progresses, long-term memory deficits become more pronounced, as are disruptions in executive function and the emergence of neuropsychiatric symptoms [115]. At a histological level, AD is characterized by neuronal loss, the appearance of reactive astrocytes and microglia, the accumulation of intracellular hyperphosphorylated Tau-based neurofibrillary tangles and the accumulation of amyloid-beta (Aβ) within the extracellular space. Notably, the appearance of AD biomarkers can occur over an extended period prior to initiation of cognitive impairment, with reports showing cerebrospinal fluid Aβ42 can precede the first signs of cognitive impairment by over 10 years, whereas abnormal levels of tau are detected shortly before the first signs of cognitive impairment [116, 117].

Another key feature of AD is the dysregulation of the circadian timing system, which is best embodied by a disruption in the sleep/wake cycle (e.g., highly fragmented and shifted sleep patterns: [118–121]), which has been reported in the preclinical phase of AD and is a well-characterized component of mid- and late stage AD [122–124]. Additionally, alterations in the clock-regulation of core body temperature rhythms, activity rhythms, the phasing of the pineal melatonin rhythm are also comorbid features of AD [125, 126].

Interestingly, several recent papers have raised the prospect that the disruption of circadian timing in AD could produce a self-reinforcing feedback loop, where disruption of timing accelerates AD pathogenesis (e.g., amyloid deposition, oxidative stress and cell death) that in turn leads to a further disruption of the circadian timing system [122, 123, 127]. The deleterious effects of this feedback loop, such as the disruption of the sleep/wake cycle and neuroinflammation, could also contribute to the cognitive deficits in AD. As outlined above, a large literature has shown that cognitive capacity is under the influence of the circadian timing system, and that the disruption of clock timing leads to marked deficits in an array of cognitive tasks [50, 128–130]. Thus, the disruption of the circadian timing system could be a key contributing factor to both AD neuropathogenesis, and the early and mid-stage cognitive impairments that are a central feature of AD.

If disruption of the circadian timing system is indeed, a contributing factor to the cognitive decline resulting from AD-a key outstanding question is centered on the identification of the location within the CNS where disruption of the circadian timing system arises. Clearly, the disruption of SCN-based timing could be pivotal; however, given the distributed nature of the circadian timing system, coupled with the broad-based disruption of cortical and limbic circuits in AD, it would not be surprising to find that alterations in timekeeping capacity within these forebrain circuits could also contribute to the disruption of cognition.

With respect to the potential connection between AD and disruption of the SCN, postmortem analysis from AD patients shows cell loss (i.e., vasoactive intestinal polypeptide-, vasopressin- and neurotensin-expressing neurons) as well as an accumulation of Tau neurofibrillary tangles: [126, 131–133]. Given the key role that both AVP and VIP signaling play in SCN timing [134, 135], one could easily envision a model wherein AD-mediated alterations of AVPergic and VIPergic signaling could lead to disrupted SCN timing and clock gated SCN output. Reactive gliosis, assessed using GFAP labeling, was also detected in the SCN of AD patients [126, 132]. Interestingly, recent work shows that clock timing within SCN astrocytes contributes to the inherent pacemaker activity of the SCN: whether a change in astrocyte reactivity could affect their capacity to contribute to SCN timing is not known [136].

In transgenic mouse models of AD, data supporting a deficit in SCN timing are somewhat mixed (comprehensively reviewed in [137, 138]). Along these lines, in the amyloid beta precursor protein-based 5XFAD transgenic line, Song et al., reported damped rhythms and alterations in the waveform of core body temperature and home cage activity at both the early stage (2 months of age) and late stage (8 months of age) of the pathological process [139]. Conversely, Nagare et al. (2020) performed a longitudinal profiling study (20 weeks to 50 weeks of age) of circadian locomotor activity in 5XFAD mice that did not detect a significant effect on the periodicity of the SCN pacemaker [140]. Further, in the 3xTg-AD mouse line, which is a mixed Aβ and tau pathology AD model, modest pathological changes were observed in the SCN (i.e., a reduction in VIP- and AVP- expressing cells) however, no effects on the free running period or light-evoked clock entrainment were observed [141]. However, in the TG4510 mouse model, which exhibits tau pathology and marked neurodegeneration, circadian profiling revealed a long free-running phenotype, tauopathy in the SCN, and damped rhythms of the core clock gene Per2, thus indicating that the molecular clock timing properties in the SCN are disrupted [142]. Collectively, the mixed result of these and other studies raises the following possibilities: 1) the inherent clock timing properties of the SCN are not markedly affected by Aβ- or tau-mediated pathologies, 2) that transgenic mouse lines do not effectively model the SCN-centric circadian disruptions observed in AD patients, or 3) that the locus of the clock disruption in AD occurs largely outside of the SCN.

Pivoting from the SCN, a number of studies have shown that oscillator populations within cortical and limbic circuits are affected in AD. In line with this idea, Cermakian et al., (2011) examined the temporal expression patterns of circadian clock genes within the cortex and the bed nucleus of the stria terminalis in postmortem tissue from AD patients [143]. Interestingly, both the phase of clock gene oscillations and phase relationships between genes and regions were altered in AD patients, relative to aged controls, thus revealing a marked temporal desynchronization of peripheral oscillators. These findings indicate that clock timing outside of the SCN is disrupted and/or desynchronized in AD patients, and in fact, the authors of this study posited that disrupted oscillatory capacity may be an independent risk factor for AD development. In addition, in the APP/PS1 AD mouse model, daily rhythms in novel object recognition memory and LTP were disrupted, and the diurnal difference in long-term spatial memory was decreased [144]. Further, in the Tg-SwDI mouse model of AD, Fusilier et al. (2021) reported a disruption in the clock-gating of spatial memory (assessed using the spontaneous alternation assay), and this decrease in clock-gated cognitive capacity was associated with a damping of molecular clock rhythms and daytime inhibitory synaptic transmission in the hippocampus [145]. When considered within the context of the noted work indicating only modest effects of AD-like pathologies on the timing properties of the SCN, these findings support the idea that the disruption/desynchronization of oscillator populations within cortical and subcortical regions, could be a key event that underlies early and mid-stage learning and memory deficits in AD. Interestingly, Kress et al. (2018) reported that the disruption of peripheral clock timing in the CNS led to an increase in ApoE expression and fibrillar Aβ plaque formation (of note however, several other measures of Aβ load did not appear to be markedly affected by the disruption of peripheral clock timing) [146].

### Chronotherapeutic approaches to improve cognition in Alzheimer’s disease

Given the mounting evidence that the dysregulation of the circadian timing system(s) is a key feature of AD, considerable effort has been invested in developing chronotherapeutic approaches for the treatment of AD. These efforts have been centered on a number of strategies that target cortical, limbic and/or SCN oscillator populations, and are designed to enhance cellular oscillator entrainment, synchronization, or to strengthen cellular oscillatory capacity; The results of these effects on cellular timing would be to enhanced clock-gated physiological output. Here we will discuss several current and potential chronotherapeutic approaches that likely function by facilitating clock entrainment, cellular clock synchronization or rhythm amplitude.

#### Early morning light therapy

To date, evidence supports the idea that light therapy, and in particular, light treatment during the early part of the day, leads to a stabilization of rhythms (as assessed by melatonin onset), sleep consolidation, and improved cognition in patients with AD [147–150]. These effects are thought to result in part from the powerful entraining effects of light on the SCN master clock. At a cellular and systems level, stabilizing SCN clock entrainment would ensure that the master clock maintains the correct phase relationship with the 24 h day. Further, stable SCN clock entrainment would also likely lead to more robust SCN rhythms; in turn, an improvement in both SCN entrainment and rhythm strength would likely be reflected in enhanced melatonin rhythms as well as an improvement in sleep quality and in cognition.

#### Gamma frequency light treatment

Gamma-band activity (~ 30–80 Hz range) is thought to facilitate effective connectivity/coherence between brain regions and correlate with attention, learning and memory retrieval [151, 152], and the circadian timing system appears to influence the frequency of gamma burst activity within cortical and limbic brain regions [153–155]. Of note, in AD patients, and in mouse AD models, there is a reduction in cortical and hippocampal gamma power activity [149, 152, 156–158]. Interestingly, in animal models, exposure to a 40 Hz light stimulus, which facilitates gamma-wave entrainment, has been shown to improve cognition and decrease AD-like pathological markers, potentially via a mechanism in which light stimulates microglia-mediated A*β* uptake [149, 159]. The precise mechanisms by which 40 Hz light therapy enhances cognitive capacity is not known; however, given 1) the role of the circadian clock in the gating of daily gamma power, 2) the reduction in gamma power in AD, and 3) the disruption in cortical/limbic circadian rhythms in AD, it is reasonable to posit that the gamma frequency therapy could be working, in part, by strengthening the circadian rhythm generating capacity of telencephalic oscillator populations. Current work is focused on testing the safety and efficacy of gamma-wave entrainment procedures on AD patients [160–162].

#### Pharmacotherapeutic approaches to enhance cellular timing in AD

With respect to AD, there are a number of cellular signaling processes that could be targeted to enhance entrainment, synchronization and/or increase the robustness and periodicity of core clock rhythms. Focusing on resetting, and thus resynchronization, of cellular oscillator populations, the ability to selectively regulate protein kinase pathways, including the p44/42 MAPK pathway, as well as signaling via Ca^2+^/calmodulin kinases, which serve as key conduits to the clock, could prove to be an effective approach [114, 163, 164]. The resetting efficacy of the noted pathways appears to be mediated, in large part, via the induction of CREB-mediated transcription, which, in turn triggers *period* expression [165–167]. As a state variable of the core clock timing mechanism, the induction of *period* gene expression would drive the resetting, and in turn, the resynchronization of cellular oscillator populations. Consistent with these ideas, numerous studies have shown that rapid clock cell synchronization can be achieved via the transient activation of the p44/42 MAPK cascade [164, 168–170]. Another potential strategy by which to entrain extra-SCN oscillator populations would be to target GRE-mediated transcription. As discussed above, signaling via GC appear to be a principal route through which the SCN sets the phasing and/or contributes to rhythmicity of peripheral oscillator populations [53, 56, 57, 171]. Here it is worth noting that signaling via both the aforementioned kinase pathways, as well as adrenal corticosteroid output, are markedly altered in AD patients, as well as in animal models of AD [172–175]. To our knowledge, the utility of chronotherapeutic strategies to target these pathways in AD (or in animal models of AD) has not been reported.

Targeting of GSK3 as a treatment for AD has been the focus of a number of investigations, given that chronically high levels of GSK3 activation have been detected in AD, and that elevated levels of GSK3 activity lead to pathogenic hyperphosphorylation of Tau protein (and in turn the deposition of neurofibrillary tangles), Aβ production, and marked cognitive deficits [176–180]. Further, in preclinical animal studies, pharmacological approaches designed to reduce GSK3 activity have been shown to reduced AD-like pathology, including Aβ production, tau hyperphosphorylation and the associated cognitive impairments [181–183]. Given the daily rhythm in GSK3 activity, and the noted roles that GSK3 signaling plays in clock timing and clock gated cellular plasticity, GSK3 may prove to be a nodal point between AD pathogenesis and the associated dysregulation of the circadian clock timing mechanism. As such, studies that examine the efficacy of GSK3 inhibitors to ameliorate clock dysregulation in AD is highly merited.

With respect to clock amplitude and periodicity, recent work has revealed that casein kinase 1 (CK1) ε/δ may be a suitable target for the therapeutic intervention against the cognitive effects of AD. Notably, daily administration of the CK1 ε/δ inhibitor PF 670462 has been shown to have profound effects on clock timing: increasing the period of the SCN oscillator and restoring rhythms in animals with disrupted/damped oscillations [13]. With respect to AD, a recent set of studies using the 3xTg-AD mouse model found that the daily/timed administration of PF 670462 rescued working memory (assessed using the spontaneous alternation assay) and re-established the capacity of the circadian timing system to drive rhythmic regulation of the hippocampal transcriptome [184]. Further, SCN-clock gated rhythmic output (assessed using locomotor activity) was normalized with daily PF-670462 treatment. Interestingly, a follow-up study found that PF-670462 led to a dose-dependent reduction in Aβ levels, and plaque size within the prefrontal cortex and hippocampus [185]. Further work will be needed to determine whether the cognitive effects of CK1 inhibition in the AD mouse models are a result of the reestablishment of clock timing within the SCN, cortico-limbic circuits, or within both regions. Nevertheless, these studies raise the prospect that targeting CK1ε/δ could prove to be a viable therapeutic strategy to address disruptions of the circadian timing system in AD patients.

Targeting the REV-ERB/ROR pathway may also prove to be an effective strategy for the treatment of AD. Support for this idea comes from recent work showing that the inhibition of REV-ERB, either through the use of the selective REV-ERB antagonist SR8278 or the genetic knockdown of REV-ERB led to an enhancement of microglial uptake of Aβ, a reduction in amyloid plaque levels, a reduction in markers of neuroinflammation, and the stabilization of synaptic physiology [186]. Consistent with these findings, the polymethoxylated flavone nobiletin, which directly binds RORα/γ and enhances *Bmal1* transcription, was recently found to confer neuroprotection and ameliorate cognitive deficits in animal models of accelerated senescence and AD [187–190]. At a mechanistic level, nobiletin was shown to reduce Aβ pathology, hyperphosphorylation of tau, and oxidative stress [190–193]. In addition, nobiletin treatment facilitated the activation of signaling pathways that underlie synaptic plasticity and memory formation (e.g., cAMP; PKA; ERK and CREB) [190–196]. Interestingly, many of the effects of nobiletin were shown to be mediated via a clock-dependent mechanism; hence, in ClockΔ19/Δ19 clock-disrupted mice nobiletin was largely ineffective in conferring resistance to metabolic stress [197]. Finally, these data, coupled with work showing that nobiletin enhances the robustness/amplitude of peripheral clock rhythms and has limited effects on the master clock in the SCN [197] suggests that the effects of nobiletin on AD pathogenesis result from the strengthening of clock timing in cortical and limbic circuits.

### Concluding remarks

**Fig. 3:**
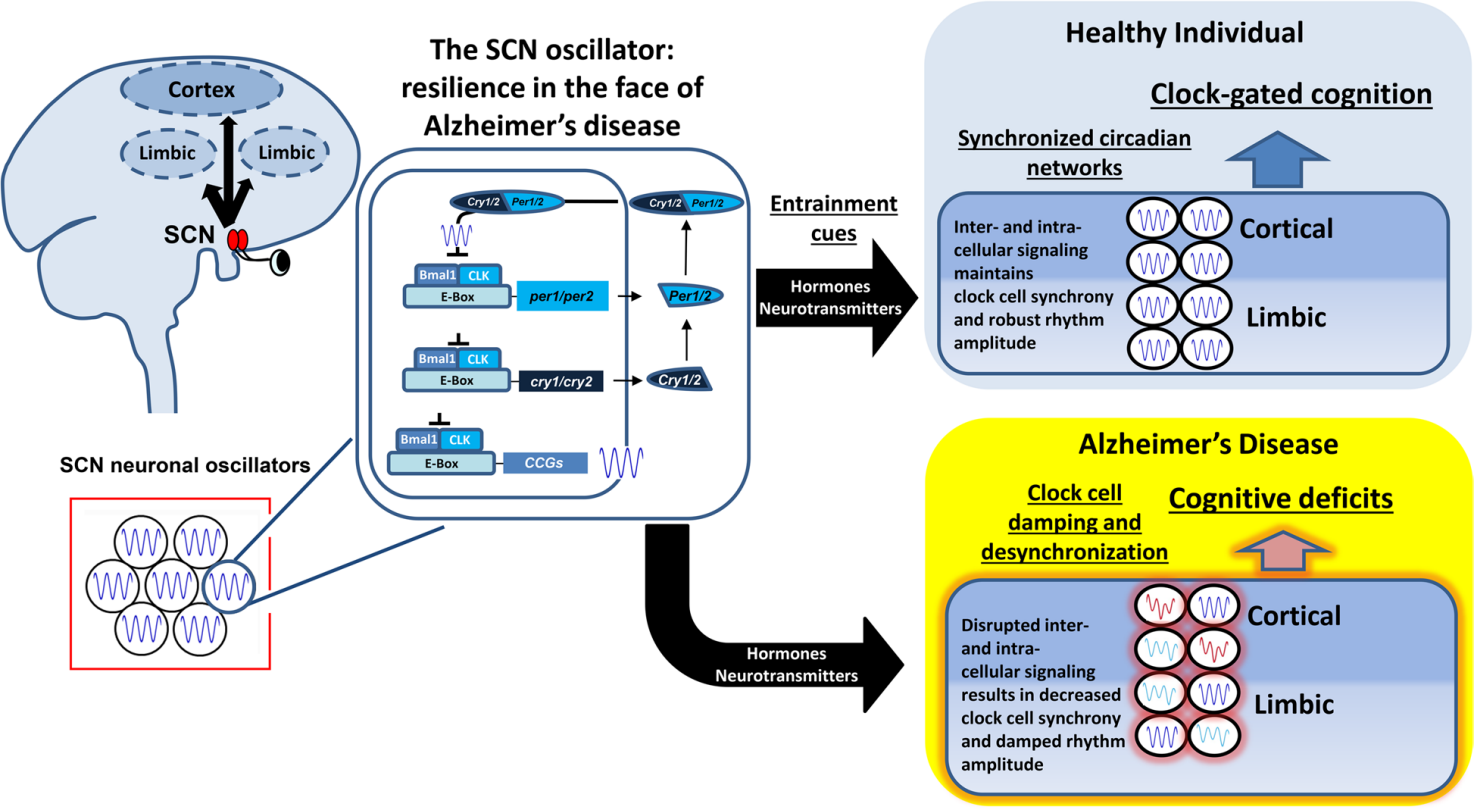
Depiction of the hypothesized process by which AD leads to a cellular and systems-level disruption of circadian timing within cortico-limbic circuits. A further description of the model is presented in the Conclusion section

A remarkable observation from several years ago is that the TTFL is cell autonomous (i.e., it does not require cellular input, and as such, it occurs in isolated cells [198]). However, for the emergence of robust and synchronized organ- and system-wide circadian oscillations, single-cell TTFL activity needs to be set to a daily phasing cue. For peripheral oscillators, these phasing cues come from the SCN (in the form of a synaptic or hormonal signal), and a loss of clock-gated SCN output (or the inability of peripheral oscillators to effectively transduce clock entrainment cues) leads to a desynchronization of peripheral oscillator populations, and a dysregulation of clock-gated physiological output [199, 200]. The work described here, suggest that early in AD-like disease progression there is a disruption of systems-level circadian timing within forebrain circuits that are required for learning and memory, and concordant with this loss of cellular timing, there are marked cognitive deficits (Fig. 3). Further, these data indicate that the fidelity of the SCN timing system is largely intact during this same time period; hence, these data raise the possibility that there is an elevated level of disruption of circadian time keeping capacity within cortical and limbic circuits (relative to the timing in the SCN), and that it is this disruption/desynchronization of forebrain oscillators from the SCN that contributes to the early- and mid-stage cognitive deficits in AD. Further studies that test key aspects of this model should provide important insights into the cellular- and systems-level circadian processes that contribute to AD.

## Data Availability

Not applicable.

## Abbreviations

Aβ: Amyloid beta
AD: Alzheimer’s disease
AKAP5: A-kinase Anchor Protein 5
ApoE: Apolipoprotein E
AVP: Arginine vasopressin
BMAL1: Brain and muscle ARNT-like protein 1 (also called MOP3)
CaMKII: Ca^2+^/calmodulin-dependent protein kinase II
cAMP: Cyclic adenosine monophosphate
CA1: Hippocampal cornu ammonis 1
CA3: Hippocampal cornu ammonis 3
CK1: Casein kinase 1
CLOCK: Circadian Locomotor Output Cycles Kaput
CNS: Central nervous system
CREB: cAMP Response Element-Binding Protein
Cry 1: Cryptochrome 1
Cry 2: Cryptochrome 2
EGR1: Early growth response 1
ERK: Extracellular-signal-regulated kinase
Fbxl3: F-box/LRR-repeat protein 3
GABA: Gamma-aminobutyric acid
GC: Glucocorticoids
GRE: Glucocorticoid responsive element
GSK3: Glycogen synthase kinase 3
KO: Knockout
LTP: Long-term potentiation
MAPK: Mitogen activated protein kinase
miR132: microRNA 132
NPAS2: Neuronal PAS Domain Protein 2
Nr1d1: Nuclear Receptor Subfamily 1 Group D Member 1
Nr1d2: Nuclear Receptor Subfamily 1 Group D Member 2
NR3C1: Nuclear Receptor Subfamily 3 Group C Member 1
Per1: Period 1
Per2: Period 2
PKA: Protein kinase A
REV-ERB: Also known as nr1d/nuclear receptor subfamily 1 group D
RORα: RAR-related orphan receptor alpha
RORβ: RAR-related orphan receptor beta
RORg: RAR-related orphan receptor gamma
RRE: Rev-ErbA/ROR response element
RSK: Ribosomal S6 kinase
SCF complex: Skp, Cullin, F-box containing complex
SCN: Suprachiasmatic nucleus
SCOP: Suprachiasmatic nucleus circadian oscillatory protein
tTa: Tetracycline transactivator
TTFL: Transcription translation feedback loop
VIP: Vasoactive intestinal polypeptide
